# Homology modeling in the time of collective and artificial intelligence

**DOI:** 10.1016/j.csbj.2020.11.007

**Published:** 2020-11-14

**Authors:** Tareq Hameduh, Yazan Haddad, Vojtech Adam, Zbynek Heger

**Affiliations:** a Department of Chemistry and Biochemistry, Mendel University in Brno, Zemedelska 1, CZ-613 00 Brno, Czech Republic; b Central European Institute of Technology, Brno University of Technology, Purkynova 656/123, 612 00 Brno, Czech Republic

**Keywords:** Homology modeling, Machine learning, Protein 3D structure, Structural bioinformatics, Collective intelligence, Artificial intelligence

## Abstract

Homology modeling is a method for building protein 3D structures using protein primary sequence and utilizing prior knowledge gained from structural similarities with other proteins. The homology modeling process is done in sequential steps where sequence/structure alignment is optimized, then a backbone is built and later, side-chains are added. Once the low-homology loops are modeled, the whole 3D structure is optimized and validated. In the past three decades, a few collective and collaborative initiatives allowed for continuous progress in both homology and *ab initio* modeling. Critical Assessment of protein Structure Prediction (CASP) is a worldwide community experiment that has historically recorded the progress in this field. Folding@Home and Rosetta@Home are examples of crowd-sourcing initiatives where the community is sharing computational resources, whereas RosettaCommons is an example of an initiative where a community is sharing a codebase for the development of computational algorithms. Foldit is another initiative where participants compete with each other in a protein folding video game to predict 3D structure. In the past few years, contact maps deep machine learning was introduced to the 3D structure prediction process, adding more information and increasing the accuracy of models significantly. In this review, we will take the reader in a journey of exploration from the beginnings to the most recent turnabouts, which have revolutionized the field of homology modeling. Moreover, we discuss the new trends emerging in this rapidly growing field.

## 1 Introduction

The protein folding problem has become an integral part of modern biology; with a historical tale that began nearly over half a century ago. Proteins are diverse heterogeneous polymers comprised of gene-coded primary sequences of amino acid monomers. Pioneering work on identification of hydrogen bond-linked protein secondary structures like α-helix by Linus Pauling and others in the 1950s paved the way to accurate experimental elucidation of atomistic (*i.e.* with fully determined xyz-coordinates for each heavy atom) protein 3D structures [1]. The use of X-ray crystallography, followed by nuclear magnetic resonance (NMR) and later cryo-electron microscopy (cryo-EM) has been dogmatic to the study of protein 3D structures in recent decades. Nevertheless, the rapid development in the field of genomics resulted in an unavoidable gap between the number of protein sequences identified and the number of experimentally validated protein 3D structures [2]. Computational methods offered a compromised solution to this dilemma. They provided faster, easier, cost-effective, non-labor intensive and practical results. The protein folding problem was approached from a thermodynamic angle (applying quantum and molecular mechanics), where the folding possibilities are scanned in potential energy conformational space (c-space) in hopes to find a state of a global minimum of energy. The computational approaches can be classified into two types of search algorithms: (1) Heuristic algorithms scan all the possibilities in c-space without *a priori* knowledge (*e.g. ab initio* modeling, Monte Carlo and molecular dynamics simulations). (2) Deterministic algorithms exclude a number of sub-spaces from c-space by utilizing *a priori* knowledge (*e.g.* homology modeling where all conformations far from the template are eliminated) [3]. In the case of homology modeling, the *a priori* knowledge is an experimental crystal structure of a template protein that is homolog to the target. In other words, a known similar protein is used to build a new atomistic 3D structure.

**Fig. 1:**
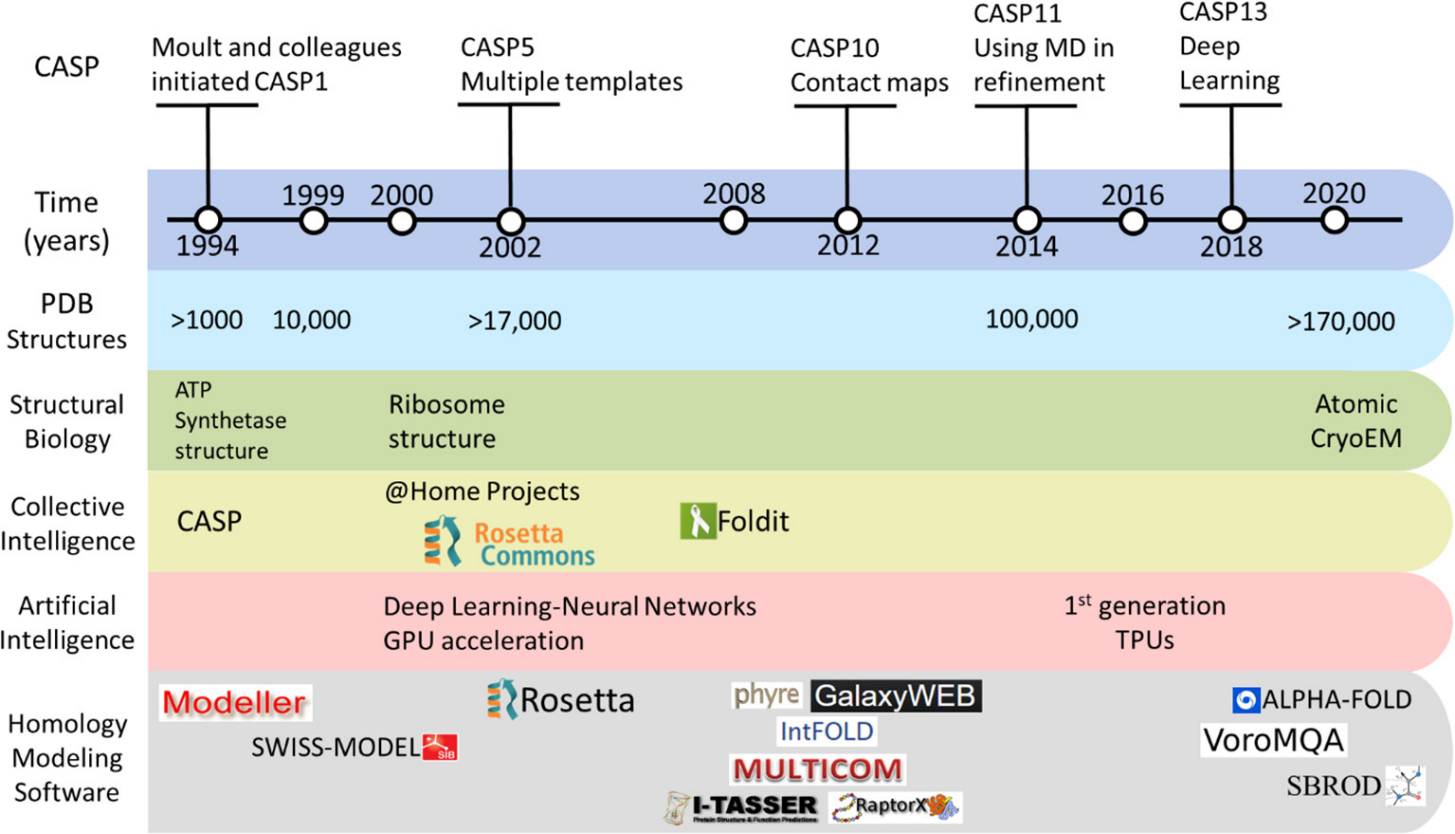
Historical timeline of major developments in homology modeling, taking into consideration the developments in collective and artificial intelligence fields. CASP: Critical Assessment of protein Structure Prediction. GPU: Graphics Processing Unit. TPU: Tensor Processing Unit.

Nearly 25 years ago, a large-scale experiment was performed for the first time to evaluate the rapid developments in protein folding prediction algorithms [4]. Until then, it was previously unknown how well protein 3D structure prediction algorithms can deliver. It was also unknown how seriously the ~35 participants and the rest of scientific community will take this experiment. Since then, the Critical Assessment of protein Structure Prediction or CASP has become a biennial event and a very well-documented record of the progress in the fields of homology modeling (TBM category or template-based modeling), *ab initio* modeling (FM category or free modeling), fold recognition and others. It was unavoidable that this experiment will shift from individual to collective intelligence (CI). Teams started to share their experiences and sooner or later what used to be biennial top-secret projects quickly became a catalyst for collaboration and development in all teams over the years. CASP and other CI initiatives will be discussed briefly in this review, covering the historic aspects of homology modeling and the lessons learned in recent years (Fig. 1). The homology modeling process is done in sequential steps where sequence/structure alignment is optimized, then a backbone is built, and later, side-chains are added. Furthermore, low-homology loops are modeled followed by optimization and validation of the whole structure.

In the past few years, the field of homology modeling was invaded and revolutionized by machine learning (ML). ML is a subfield of computer science that gives computers the ability to learn without being explicitly programmed. According to recent definitions, it is an umbrella term that refers to a broad range of algorithms that perform intelligent predictions based on a dataset [5], [6]. ML is a branch of artificial intelligence (AI) (not to be confused with data mining). AI is the capability of a machine to imitate intelligent human behavior using reason, devising strategy, solving puzzles, and making judgments under uncertainty, representing knowledge including common-sense knowledge, planning, learning, communicating in natural language and integrating all these skills towards common goals [7], [8]. On the other hand, the definition of data mining is to mine information and discover knowledge without explicit assumptions, that is, without prior research and design, the information obtained should have three characteristics: previously unknown, effective, and practical [9]. ML was recently introduced to homology modeling showing unprecedented improvements in prediction accuracy. In brief, ML includes a wide range of algorithms used for extracting certain features from data in order to perform predictions on new data. In other words, the dataset is used to estimate unknown dependencies of a system in order to predict new outputs of that system [10]. The process is done by (1) collecting and describing data, (2) building mathematical/statistical model, and (3) evaluating the model performance. The protein 3D structure dataset should be in high quality and well formatted for ML computations [11]. The model building and evaluation is often performed by dividing the data into training and testing sets for ML algorithms. These algorithms include logistic regression, decision trees, support vector machines, random forests, artificial neural networks, and many other methods [12]. From now on, we will refer to artificial (non-biological) neural networks as neural networks in the rest of the review.

## 2 Homology modeling

In one seminal review, Marti-Renom *et al.* (2000) [13] envisioned the necessity for large-scale genome-wide automated homology modeling (which used to be called comparative modeling) in order to face the torrents of new genomic sequences. The same challenges of that time, namely: “weak sequence–structure similarities, aligning sequences with structures, modeling of rigid body shifts, distortions, loops and side chains, as well as detecting errors in a model.” are still recognized till this day. Homology modeling depends on two principles: first, the primary sequence of amino acids determines the protein 3D structure, and second, the protein 3D structure is somehow conserved with regards to the primary sequence. Although that seems like an easy and direct task, nevertheless it is not; in fact, protein folding and 3D structure formation rules are not black and white. However, using homology modeling can fill the gap between primary and 3D structures, which will permit us to deduce functional and useful properties in the same way an experimental 3D structure can be applied. Thus, giving us access to more therapeutic targets and many other applications such as the study of protein function (*e.g.* catalytic enzymes and their substrates), the structural roles of proteins in the cell (some proteins serve as building blocks in the cell), and protein interactions (such as antibody binding) [2], [14], [15], [16]. Structural genomics is a broad and ambitious concept that was introduced nearly two decades ago, in which scientists hope to one day be able to determine 3D structures of all proteins encoded in the genome. Such technological advancement can answer numerous questions about cellular functions, tissue specialization, signaling pathways, and disease mechanisms. Furthermore, disease-related mutagenesis studies are another avail of homology modeling in the aspect of identification of amino acids with relevant function in a protein. Homology modeling tools are also applied in molecular modeling of biological assemblies of protein complexes (*e.g.* entire virus 3D structure), and in protein–protein interaction studies [15], [17], [18], [19]. One of the most recent applications of homology modeling is the refinement of cryo-EM 3D structures, in which computational methods are used to analyze 3D molecular surface and density maps, followed by homology modeling used to generate atomic 3D model [20], [21], [22], [23]. Recently, Single-particle cryo-EM has acquired atomic resolution, which not only enables the visualization of atoms in a protein, but also observation of density for hydrogen atoms and imaging of single-atom chemical modifications [24].

**Fig. 2:**
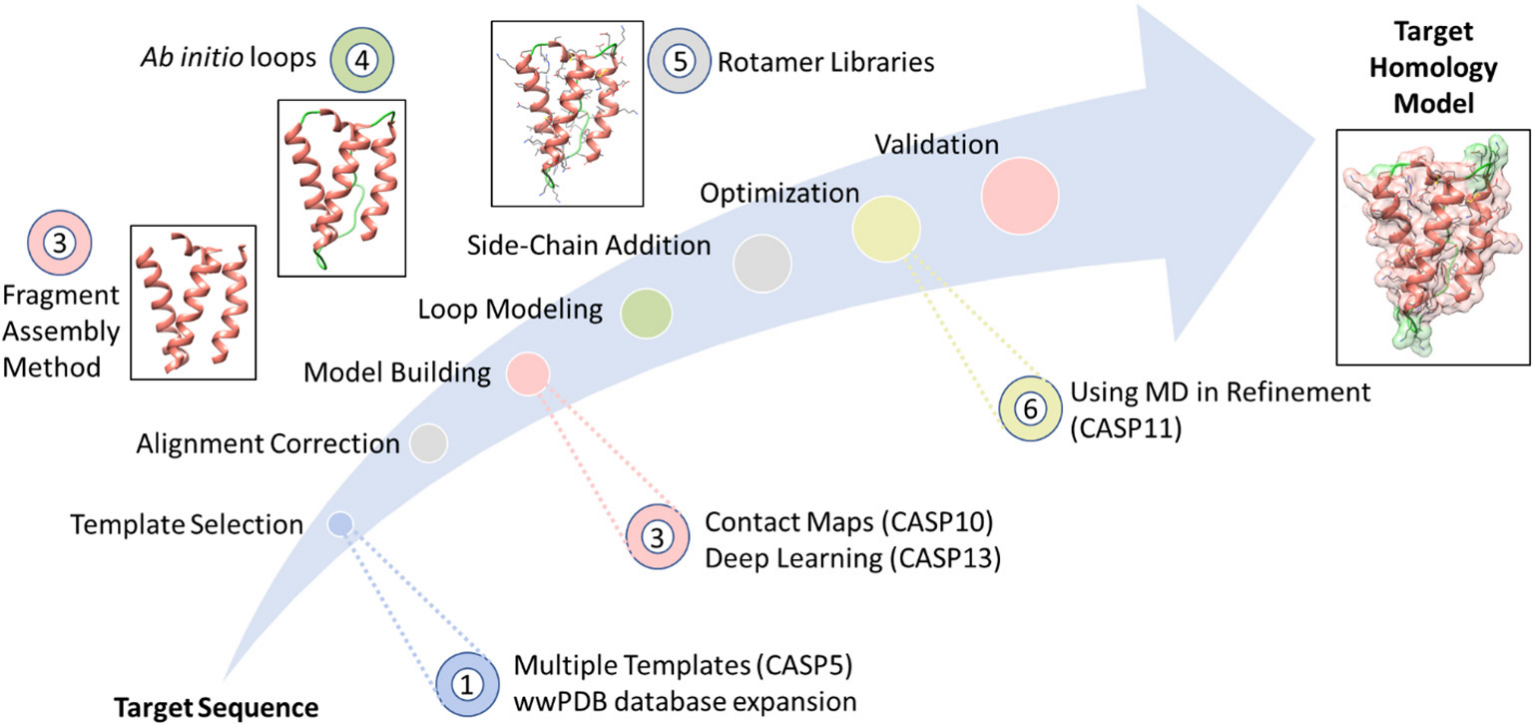
The seven classical steps of homology modeling. Donut shapes describe the major events influencing some of the homology modeling steps.

The process of homology modeling itself is run by seven classical steps (Fig. 2):

1. ***Identification and selection of templates*** (other homologous proteins with known 3D structures). Depending on the first principle, we start searching for eligible templates based on sequence–sequence alignment, while narrowing our search to the crystal structures deposited at the Worldwide Protein Data Bank (wwPDB) database (http://www.wwpdb.org/). The eligible templates are chosen using protein Basic Local Alignment Search Tool (BLASTp). In the case of low homology (below 35% sequence identity; the number of identical amino acids in an alignment), alternative methods are used for alignment to reduce shifts and gaps such as profile-profile alignments, Hidden Markov Models (HMMs) and position-specific iterated BLAST (psi-BLAST). Profile HMMs generate more accurate alignments than psi-BLAST, such as HMM-HMM–based lightning-fast iterative sequence search (HHblits; http://toolkit.genzentrum.lmu.de/hhblits/) [25], and iterative profile-HMM search method, JackHMMER [26]. Very low sequence identity will lead to false folding assignments due to alignment errors resulting from more gaps and mutations [2], [16], [27], [28], [29]. Multiple alignments (*e.g.* CLUSTALW [30], Clustal Omega [31], and MUSCLE [32]) and using multiple templates can improve the modeling process.
2. the previous step is followed by ***correction and optimization of the chosen alignments*** (usually with multiple template 3D structures) in order to build the whole backbone [29], [33].
3. ***The 3-D model building*** is then performed using one of four different approaches [2], [34]:
  i. The rigid-body assembly method collects rigid body parts together, which are picked up from the aligned template protein structures, using programs like 3D-JIGSAW, BUILDER, and SWISS-MODEL.
  ii. The segmented matching method relies on comparing the template and structures in the database, based on the sequence identity, geometry, and energy such as the SegMod/ENCAD program.
  iii. The spatial restraint method approach is another method that depends on the restrains of the template, and can be done using MODELLER.
  iv. The artificial evolution method depends on rigid-body assembly and stepwise template evolutionary mutations, and can be done using NEST.
4. The next step is ***loop modeling***. Loops sometimes contribute to important protein functions where the accuracy of loop prediction is crucial to the model whole value. Loop prediction is a complex process because loops are variable and not conserved. Loop prediction is done by two methods: the first is database search approaches which depend on comparison with all the known proteins, the second is conformational search approach (*ab initio*) which depends on scoring function optimization; a more direct approach [29], [35].
5. ***The addition of side-chains*** onto the major backbone is very critical step. This process requires selection of a rotamer library, a scoring function and a scanning method [36]. Several programs have been developed to add the side-chain rotamers, such as: OPUS-Rota2 [37], SCWRL [38], and FASPR [39] to name a few tools. We need to emphasize that most homology modeling servers and programs perform the previous steps (from input sequence to building 3D structure) in automated fashion, however many of the tools previously described can be used independently to fix errors in the model.
6. The previous step is followed by ***model optimization***, which is used for increasing the quality of the final model. This step is done by using energy minimization utilizing molecular mechanics force fields, to reduce atomic clashes, and exclude all major and small errors. Further optimization can be done using molecular dynamics and Monte Carlo simulations [40].
7. The final step is ***model evaluation and validation*** (Table 1), where the value and function of the model are correlated with the model accuracy. For this purpose Distance-matrix ALIgnment (DALI, http://ekhidna2.biocenter.helsinki.fi/dali/) or Verify3D (https://servicesn.mbi.ucla.edu/Verify3D/) can be employed. The value of the model is decided depending on the stereochemistry, physical parameters, knowledge-based parameters, statistical mechanics, and many other criteria. The ultimate model validation would be assessment against real experimental 3D structure. It is advised to use several evaluation methods simultaneously to yield the best results. One of the challenges in modeling is the reduced accuracy or production of incorrect models. Alignment errors are still the main cause of deviations and the previous challenges need careful manual inspection and adjustment even when using fully automated programs [16], [17], [41].

**Table 1:** Scores used in protein 3D structure comparison and evaluation. Distance-based and contact-based similarity scores are used in experimental evaluation of homology models. Other scores are used for quality check such as physics-based, knowledge-based and combined scores.

**Score**	**Description**	**Reference**
***Distance-based similarity scores:***		
RMSD	Root mean square deviations	
wRMSD	weighted RMSD	
RMS of dihedral angles	Root mean square of dihedral angles	[42]
GDT	Global distance test employing local global alignment (LGA) program	[43]
GDT_TS	GDT total score: Iterations superposing sets of 3, 5 and 7 consecutive Cα atoms (thresholds 1, 2, 4, and 8 Å)	[44]
GDT_TL/GDT_HA	GDT high accuracy scores: Finer thresholds than GDT_TS (0.25, 0.5, 1, and 2 Å)	[14], [44]
TM-score	Variations between Cα atoms weighting residues at shorter distances	[45]
TM-align	Based on TM-score for evaluating global variations	[45]
MaxSub	Normalized score from large subset of Cα atoms	[46]
SphereGrinder	Specialized for large predicted models	[47]
***Contact-based similarity scores:***		
CAD	Contact area difference	[48]
CAD-score	Contact area difference	[49]
***Physics-based quality scores:***		
Molprobity score	Global (whole protein) and local (small regions) perspectives	[50]
What IF	Surface area, solvent accessibility, and hydrophobicity checks	[51]
PROCHECK	PROgram to CHECK stereochemical quality	[52]
***Knowledge-based quality scores:***		
QMEAN	Qualitative Model Energy ANalysis	[53]
DOPE	Discrete Optimized Protein Energy	[54]
PROSAII	PROtein Structure Analysis II	[55]
***Combined quality scores:***		
MetaMQAP	Meta-methods for quality assessment of protein models	[56]
Machine learning methods	Using support vector machine (SVM) to combine scores	[57]
Z-score	Any arbitrary score function based on sum of a number of scores (*e.g.* force field energies, GDT, *etc*.) May or may not be normalized	
***Other methods:***		
Experimental studies	Validation can be done by other experimental data, such as molecular dynamics simulations, spectroscopic methods, binding analysis (*e.g.* calculations of dissociation/inhibition or Kd/Ki constants)	[58], [59]

## 3 Homology modeling programs

**Fig. 3:**
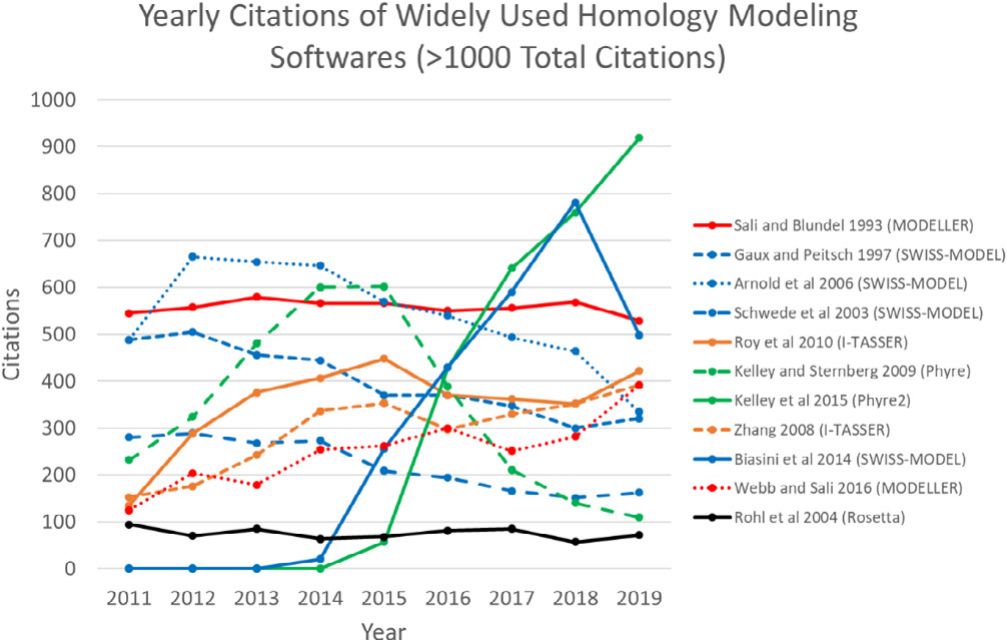
Yearly citations of widely used homology modeling programs (defined as those having > 1000 total citations). MODELLER (red) and SWISS-MODEL (blue), which date over two decades are the most popular among researchers, whereas the popularity of I-TASSER (orange) and Phyre2 (green) is on the rise (source: webofknowledge.com). (For interpretation of the references to colour in this figure legend, the reader is referred to the web version of this article.)

Various molecular graphics programs are used for visualization and editing of protein 3D structures (Table 2). The number of fully automated homology modeling programs has been growing. However, for the past three decades, few programs have been ever increasing in popularity at a steady pace (Fig. 3).

**Table 2:** Molecular graphics programs.

**Program**	**URL**	**Reference**
Avogadro	http://avogadro.cc/	[60]
DeepView (SwissPDB Viewer)	https://spdbv.vital-it.ch/	[61]
EzMol	http://www.sbg.bio.ic.ac.uk/ezmol/	[62]
Jmol	http://jmol.sourceforge.net/	[63]
MOE (Molecular Operating Environment)	https://www.chemcomp.com/	[64]
Molden	https://www3.cmbi.umcn.nl/molden/	[65]
PyMOL	https://pymol.org/2/	[66]
RasMol	http://www.rasmol.org/	[67]
SAMSON	https://www.samson-connect.net/	[68]
Scigress	https://www.fqs.pl/en/chemistry/products/scigress	[69]
UCSF Chimera	https://www.rbvi.ucsf.edu/chimera/	[70]
VMD (Visual Molecular Dynamics)	http://www.ks.uiuc.edu/Research/vmd/	[71]
WHAT IF	https://swift.cmbi.umcn.nl/whatif/	[72]
YASARA	http://www.yasara.org/	[73]

MODELLER [74], [75] is a program inspired by similar techniques used in NMR structure determination called modeling by satisfaction of spatial restraints. Using probability density functions, these restraints/parameters are combined in one objective function that is minimized by conjugate gradient and molecular dynamics with simulated annealing. These restraints include: homology-derived restraints on the distances and torsional angles in the query sequence/template structures alignment; stereochemical restraints such as bond length and bond angle parameters obtained from a force field; parameters for torsional angles and non-bonded interatomic distances; and finally optional restraints, such as those from experimental data.

SWISS-MODEL [76], [77], [78], [79] is an automated server with minimal user input, usually in the form of primary sequence. Templates are selected and aligned from an extracted database (exPDB), and models are built for all regions except insertions and deletions in the target-template alignment. The gaps are built using constraint space programming to select the best loop. A backbone-dependent rotamer library is used to add the side-chains and the model is optimized by steepest descent.

I-TASSER [80], [81] is an iterative threading assembly refinement server used to generate homology models from multiple threading alignments and iterative structural assembly simulations. The target sequence is matched against a non-redundant sequence database by psi-BLAST tool to identify homologs. A sequence profile is also used to predict the secondary structure. It is then threaded through a representative 3D structure library using LOMETS tool to rank the templates for further consideration. The threads are assembled and the loops are predicted by *ab initio* methods. Models are generated using a modified replica-exchange Monte Carlo simulation and the top cluster is selected using SPICKER tool, and the model is finally optimized and evaluated.

Phyre [82] and the updated Phyre2 [83] are servers that use advanced remote homology detection methods to build 3D models, predict ligand binding sites and analyze the effect of amino acid mutations. Psi-BLAST and secondary structure prediction algorithms are used to align the target sequence on template 3D structure. The scan of 20% identity non-redundant database is curated using HHblits to create multiple sequence alignments, which are later used to predict secondary structure with PSIPRED. Both the alignment and secondary structure prediction are combined into a query HMMs. The best alignments are used to build a model from a database of known 3D structures HMMs. Finally, the loops are modeled and side-chains are added accordingly.

Rosetta [84] is a program based on *de novo* structure prediction algorithm, yet it is used for protein folding in divergent domains of homology models. Initial protein folding of short segments is chosen from the protein 3D structure database, whereas longer segments are constructed using 3 and 9-residue fragments selected from the database and combined using the Rosetta algorithm.

RaptorX [85] is a server, which was developed by addition of several enhancements on the previous RAPTOR program. First, the quality of sequence profiles is assessed by a profile-entropy scoring method that considers the available non-redundant homologs. Second, conditional random fields are used to integrate a variety of biological signals in a nonlinear threading score function. Multiple-template threading tool allows for the use of multiple templates to model a single target sequence, which can correct some errors in pairwise alignments.

GALAXY, GalaxyTBM [86] or GalaxyWeb [87] is a server which employs HHsearch and PROMALS3D tools for template selection and sequence alignment. The core regions are retained while the unaligned regions are removed and later added in refinement using *ab initio* loop modeling. The model is globally optimized by conformational space annealing in which a maximum of three unreliable local regions are reconstructed.

AlphaFold [88], which is developed by DeepMind company, relies more on *ab initio* modeling principles. Here, co-evolutionary analysis is used for matching amino acid sequence co-variation with physical contact in protein 3D structure, and later, these maps are studied using deep neural networks to identify patterns in protein sequence and co-evolutionary couplings and convert them into contact maps. The approach can be considered a modification on RaptorX modeling. RaptorX uses multiple sequence alignments to predict probabilities of discrete distances (mean and variance) to limit the atom–atom distances in predicted ranges that are used to feed geometric constraint satisfaction algorithm. Unlike RaptorX, AlphaFold exploits the entire probability distribution in a continuous function, which is later minimized.

## 4 Independent evaluation experiments

Due to growing number of programs and tools used in homology modeling, several research groups attempted to benchmark and evaluate the homology modeling programs independently. In a benchmarking experiment, Wallner and Elofsson [89] evaluated six homology modeling programs, namely: MODELLER, SegMod/ENCAD, SWISS-MODEL, 3D-JIGSAW, NEST, and Builder. Among these, MODELLER, NEST, and SegMod/ ENCAD were the best performers. Similarly, Dalton and Jackson [90] evaluated five homology modeling programs (Builder, NEST, MODELLER, SegMod/ENCAD and SWISS-MODEL) using three alternative sequence-structure alignment programs (3D-Coffee, Staccato and SAlign). Their findings showed MODELLER to be the best performer among these. Forrest *et al*. [91] used MODELLER to evaluate the accuracy of numerous types of alignments in predictions of homology models of membrane proteins, such as sequence-to-sequence alignments (*e.g.* ClustalW), sequence-to-profile alignments (*e.g.* Psi-BLAST of each template then align queries with ClustalW), Multiple-sequence alignments (*e.g.* Psi-BLAST followed by ClustalW, T-Coffee, MUSCLE and ProbCons), profile-to-profile alignments (*e.g.* HMAP) and structure-based alignments (*via* SKA). For identities>30%, their findings showed that profile-to-profile alignments produced the best homology models. Despite their thoroughness and comprehensiveness, the field of homology modeling is rapidly growing beyond a single evaluation experiment. Experimental 3D structure repositories are growing, while homology modeling programs and servers are updated continuously.

## 5 Collective intelligence

The Wikipedia defines CI as “shared or group intelligence that emerges from the collaboration, collective efforts, and competition of many individuals and appears in consensus decision making” [127]. In their attempt of formulating the first mathematical CI definition, Szuba *et al*. [92] identified CI as distinguished concept from individual and artificial intelligences, thus generalizing the mathematical definition to be applied on bacteria, other organisms, and even inter-species groups. Such formalism accepts collective resources and software programs as entities communicating (interacting) within the collective. Again in their generalized mathematical formalism, Szuba *et al*. [92] argued that “in a socially cooperating structure, it is difficult to differentiate thinking and non-thinking beings (abstract beings must be introduced)”. Nevertheless, more community-oriented intelligence concepts were trending in the past few decades. Wisdom of the Crowd is a concept, which attributes the best judgments to the ones made by a group of people as compared to the ones made by the best person in the group. The value of this phenomenon lies in being able to sift the noise out in individual judgments in order to get closer to the ground truth using the clear voice of the group. This concept can be applied on the synergism of the scientific communities or research groups [93]. In contrast, Crowd-Sourcing is a problem-solving strategy, which involves an organization having a large group of people attempting to solve a problem or part of a problem then sharing the solutions. This strategy allows large groups of individuals to practice wisdom of the crowd by participating in research projects through innovation-challenges, hackathons, and related activities, which can eventually achieve faster and more efficient outcomes [94], [95]. The ultimate level of merging CI and AI is called Symbiotic intelligence (SI). From a biological aspect, symbiosis is a beneficial relationship between two organisms living together, however, in the aspect of bioinformatics definition, SI is the new paradigm of co-operation between humans and computers to perform more advanced applications combining the much computational breadth of the human brain with the much computational depth of the computer processers [96], [97].

## 6 Collective intelligence and protein folding

Numerous developers of popular homology modeling programs/servers (*e.g.* MODELLER, SWISS-MODEL, I-TASSER, *etc*.) established huge database repositories of homology-modeled 3D structures and supplemented them with prediction algorithms for annotation of secondary structures, protein domains and functions. No one can deny the role of CASP in motivating the scientific community for active development of the homology modeling field, although more in some years than others as detailed in the next section. CASP inadvertently contributed to the development of homology model evaluation techniques. It is also clear that developments in *ab initio* modeling (FM category in CASP) have also inspired the development of homology modeling indirectly through initial contact maps and directly through *ab initio* loop modeling tools, which are integral part of many homology modeling programs. We have used the term “collective intelligence” to describe the situation when a group of researchers is working, dependently or independently, competitively or uncompetitively, actively or passively, towards a unified goal. Here, we will describe three other examples of CI that shaped our perception of the protein folding problem (not only in homology modeling), namely: @home projects, RosettaCommons and Foldit.

@Home projects are distributed computing projects that motivate volunteers by giving them certified Berkeley Open Infrastructure for Network Computing (BOINC) credits. There are nearly 40 BOINC projects at the moment. Folding@Home (https://foldingathome.org/) is a distributed computing project that utilizes the volunteers’ computational resources such as CPU power, disk space, and network bandwidth. The project, which started in 2000, used molecular simulations to study the folding and functions of many proteins, in some cases for over 1.5 ms time scale, and published nearly 225 articles. It is estimated that nearly 4 million personal computers around the world are participating in this project and competing to earn points. In their perspective entitled “Screen Savers of the World Unite!”, Shirts and Pande [98] argued for the utilization of unused CPU-time in a period where computational costs for molecular simulations were extremely expensive. This project describes a dependent, competitive and passive form of CI. Rosetta@Home (https://boinc.bakerlab.org/rosetta/) is another distributed computing project from David Baker’s lab that was announced in 2005, and currently holds over 53,000 active volunteers from 150 countries. Predictor@home is an example of another distributed computing project to predict 3D structures using dTASSER. However, it was discontinued in 2009 [99]. The Human Proteome Folding Project (HPF) [100] is another discontinued distributed computing project on the World Community Grid (WCG, developed by IBM company), which utilized Rosetta and was active in the years 2004–2013.

RosettaCommons (founded in 2001) is an example of a collaborative initiative of > 500 developers that began in the mid-1990 s, where the scientific community is sharing a codebase for development of computational algorithms [101]. Eventually, this library of over 3.1 million lines of code have grown to become one of the largest programs in molecular modeling. RosettaCommons describes a dependent, uncompetitive and active form of CI that was able to avoid the fate of many old programs by establishing sustainable, ever-growing and well-maintained CI.

Another unique initiative is the Foldit project (http://fold.it/), also developed in 2008 by David Baker’s lab at the university of Washington [102]. Foldit is a protein folding puzzle video game, which can be viewed as a clear example for the role of competitiveness and crowd-sourcing in prediction of protein 3D structures. Recent successes of Foldit project highlight the applications of this video game in *de nov*o protein design (*viz.* synthetic biology), which were validated by the study of the designed structures using NMR and cryo-EM [103], [104].

## 7 Collective intelligence and the CASP experiments

The protein folding problem still remains one of the most important questions in biology. What controls the speed of folding and why does a protein choose a certain folding state? Furthermore, can we design a logical algorithm that predicts the 3D structure and its changing dynamics using amino acid sequence alone [105]? With regards to the third point, Moult and colleagues initiated an experimental event that is held every second summer. CASP started in 1994 by sending invitations to the known researchers in the field and by advertisements in journals. Different teams participated worldwide to predict different protein 3D structures using their algorithms. The results of the different teams were then compared with the true experimental structures in a “Blind” prediction regime. The organizers provided all the teams with the same protein sequence targets, with balanced range of difficulty, in order to catch a panoramic view of the modeling problems of that time [106], [107], [108]. The CASP experiment can be viewed as a unique scientific sociological structure; an approach to advance protein science through organized, collaborative and communal effort. The protein folding question was no longer a one individual problem, but rather a complicated field which requires enormous efforts to move one step ahead [105]. The age of the CASP is 26 years and still counting. In the first decade of the experiment, prediction accuracy has improved positively from year to year with steady yet modest progress from CASP1 to CASP6. This can be explained by the expansion of the PDB database (Fig. 2) and the emerging of new sequence search and alignment tools such as BLAST. Further, the emerging of the fragment assembly method after CASP4 gave the researchers the ability to treat each identifiable domain as a separate target; which also left a positive imprint on the first decade’s results [109], [110]. In CASP6, a new measurement, also known as the global distance test GDT_TL parameter (Table 1), was used along with the old less sensitive GDT_TS parameter to show the slight accuracy improvement between CASP5 and CASP6, in addition to improved server performance [110]. CASP7 introduced two major changes: The first change was in closing the accuracy gap between the human and automated server in terms of prediction; an important step towards high throughput modeling. The second change was model choice based on a single best template structure to predict the performance. Overall, the progress from CASP6 to CASP7 has been sustained in the mid-range difficulty targets. However, at that point there were still challenges in the prediction of large complex molecules, *ab initio* modeling and refinement techniques [111].

At the end of the second CASP decade, more difficult targets were introduced, while the development of multiple template methods and small single-domain *ab initio* structures modelling have advanced. Two encouraging developments in CASP10 edition were the development of new refinement methods and the improved methods of predicting contact maps (defined by residue-residue proximity based on threshold distance between the Cβ atoms of the side-chains) [112]. The CASP11 experiment achieved few things that were expected from the last optimistic edition: the improvements were in the new contact map methods of the *ab initio* models, the refinement methods using molecular dynamics for estimating the accuracy of models, and finally modeling of non-principal templates regions [113].

CASP12 edition revealed acceleration in the progress of contact accuracy using new methods for predicting residue-residue contacts, as well as *ab initio* modeling due to these developed methods. The newly available data for protein sequences and 3D structures from many resources contributed to these previous improvements. The torrents of data have assisted modeling by combining experimental and computational forces together. Many research teams relied on refinement using molecular dynamics. This CASP and the previous one established a new assessment to decide whether a model is adequate for answering a particular biological question. A new assessment category for protein assembly was also added [114].

**Fig. 4:**
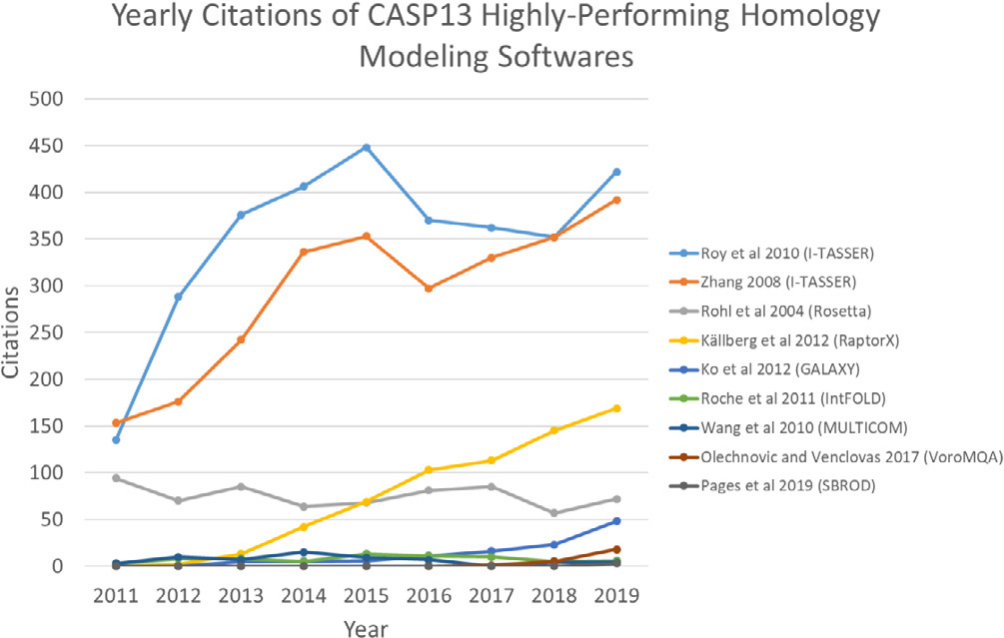
Yearly citations of CASP13 highly-performing homology modeling programs. I-TASSER and RaptorX are the most rising in popularity among researchers (source: webofknowledge.com).

By far, the CASP13 had the most dramatic changes, starting with the changes in target composition from *ab initio* and homology models due to progress in resolution of cryo-EM. A striking development was seen in backbone accuracy as a result of the effective deployment of deep ML methods. The most surprising improvement was spotted in *ab initio* modeling, one of the toughest aspects of the experiment, as a reflection of the progress in contact prediction. Homology modeling displayed superior results from CASP12 due to the impact of deep ML methods and contact prediction. Overall, the most two general features that made this total difference are the introduction of a new formulation of contact prediction and new deep network architecture [115], [116]. Amongst the highest performers in CASP13, it is clear that I-TASSER and RaptorX were already growing in popularity among researchers, while two highly sophisticated and accurate performers were emerging rapidly, namely GALAXY and AlphaFold (Fig. 4 and Table 3).

**Table 3:** Top performing homology modeling programs in CASP13.

Rank	Program/Server	Year	Country	Interface	Website	References
1,3,5	I-TASSER	2008	USA	Server (C++)	https://zhanglab.ccmb.med.umich.edu/C-I-TASSER https://zhanglab.ccmb.med.umich.edu/C-QUARK/	[80], [81], [117]
2,11	GALAXY	2012	South Korea	Server (Python)	http://galaxy.seoklab.org/	[86], [87], [118]
4	AlphaFold	2019	UK	Package (Python and C++)	https://github.com/deepmind/deepmind-research/tree/master/alphafold_casp13	[88], [119], [120]
6	IntFOLD	2011	UK	Server or Package	https://www.reading.ac.uk/bioinf/IntFOLD/	[121]
7,9	RaptorX	2012	USA	Server or Package	http://raptorx.uchicago.edu/	[85], [116]
8	VoroMQA	2017	Lithuania	Server	http://bioinformatics.ibt.lt/wtsam/voromqa	[122]
10	SBROD	2019	France	Server or Package (C++ and Python)	https://gitlab.inria.fr/grudinin/sbrod	[123]
12	MULTICOM	2010	USA	Server or Package	http://sysbio.rnet.missouri.edu/multicom_cluster/	[124], [125]
13	Rosetta	2004	USA	Server or Package (C++)	https://www.rosettacommons.org/home	[84], [126]

## 8 Artificial intelligence and protein folding (From Machine learning to deep Learning)

Since the mid-1990 s, different computational algorithms were involved in protein secondary and tertiary structure prediction such as genetic algorithms, graph theory, ML and neural networks [128]. While many of the conventional ML methods can be substituted with other statistical methods, the main trigger to catalyze the use of deep learning neural networks in homology modeling was the dawn of “big data” era. The rising momentum in protein sequence and structure data production came from both computational and experimental sources. Within ten years, the applications of contact maps and the need to apply complete contact distance distributions instead of discretized data were increasingly envisioned to lead the way to more accurate 3D structure predictions.

**Fig. 5:**
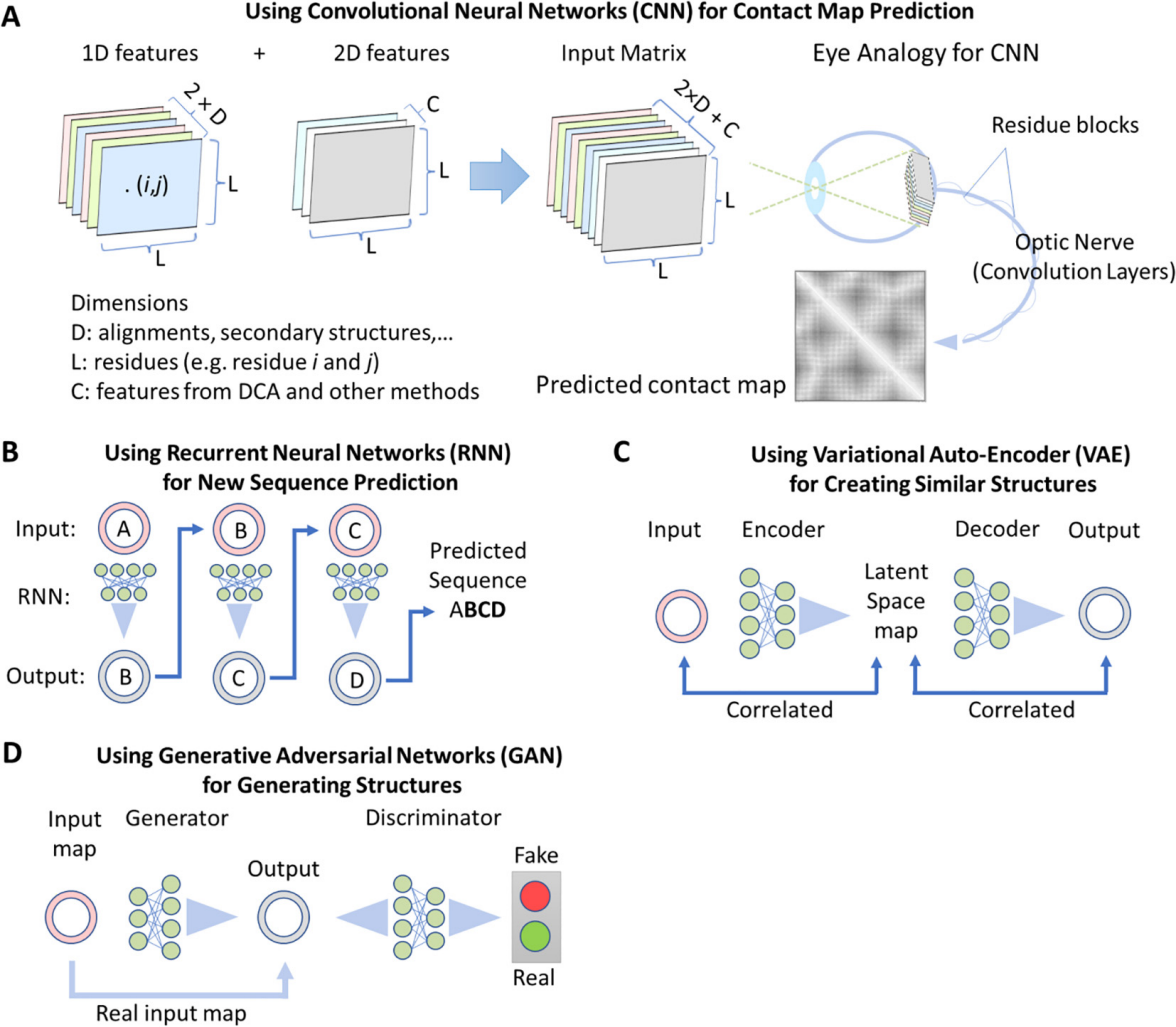
Neural network strategies used in protein 3D structure prediction tools (as described in [133], [139]). (A) Convolutional Neural Networks – CNN are employed by merging 1D features and 2D features dimensions into residue blocks that are used as input matrix for convolutional layers. Just like an optic nerve, the residue blocks are convolved into smaller and smaller layers. (B) Recurrent Neural Networks – RNN are trained for generating sequences. (C) Variational Auto-Encoder – VAE is used for creating similar structures that are correlated with an input structure. Properties are calculated by constructing a latent space map, which is then used to produce outputs. (D) Generative Adversarial Networks – GAN use a gaming method to discriminate real input from fake input that is produced from a generator. The game continues until the discriminator is unable to distinguish the real from fake outputs.

Deep learning convolutional neural networks (CNN; Figure 5**A**) in protein structure prediction were recently reviewed by Torrisi *et al*. [129]. Two classes of protein structure annotations (PSA) predictions were used to identify the deep learning tools used, namely 1D and 2D features. 1D-PSA included models to predict secondary structures, solvent accessibility, torsion angles, contact density and disordered regions. 2D-PSA included models to predict distance maps (*e.g.* AlphaFold), multi-class contact maps (*e.g.* DeepCDpred [130] and RaptorX-Contact [131]) and contact maps (*e.g.* I-TASSER’s TripletRes [132] and the rest of modeling tools). In another recent review, Gao *et al*. [133] described four common strategies of deep learning neural networks that can be applied in protein 3D structure prediction:

1. CNN are widely used in image analysis and most widely used in protein 3D structure prediction [129], *e.g.* RaptorX and AlphaFold. They are based on convolutional kernels where the input passes through convolutional layers. The inputs are convolved (coiled or rolled) in a restricted region just like in a biological system when cortical neurons respond to stimuli only in a restricted region of the visual field (Figure 5**A**).
2. Recurrent neural networks (RNN; Figure 5**B**) are widely used in sequence data such as text and time series, and they learn in a sequential (*i.e.* autoregressive) way. Therefore, their best application would be for protein sequence generation or prediction of the next amino acid at the terminus of a protein.
3. Variational auto-encoder (VAE; Figure 5**C**) is an example of unsupervised learning method. Unlike the previous neural networks, VAE does not predict a new output, but rather learns new and simpler representation (map) of the original input through an optimization method called variational inference. VAE can take the protein 3D structure and learn certain properties (by constructing a map called latent space). This map is correlated with protein 3D structure properties. Convolutional VAE was previously used for clustering of protein folds from molecular simulations [134]. Obviously, this method has potentials for design of similar protein/peptide 3D structures that have similar properties [135].
4. Generative adversarial network (GAN; Figure 5**D**) is a gaming method between two adversaries: a generator and a discriminator. The former generates a map of a distribution input (*e.g.* Gaussian), while the latter tries to learn if it is real or fake. The process of learning by the two adversaries continues by stochastic optimization until an equilibrium is reached. This strategy have been successfully applied in loop modeling [136], and for generating torsional angles [137], protein backbone models and 3D structures [138].

It is important to emphasize that contact maps often contain transitive noise coming from indirect correlations between residues [139]. Methods for direct correlation analysis are used to remove this noise such as Direct Coupling Analysis (DCA), Protein Sparse Inverse COVariance estimation (PSICOV), and network deconvolution (ND). In modeling a sequence generator, DCA can be used to calculate the probability of each generated sequence by estimation of a partition function. Several DCA partition function estimation techniques have been developed and applied in contact map prediction (Table 4). On the other hand, PSICOV depends on the principle of partial correlations, where you calculate the correlation between two elements while excluding the influence of a third element. ND and balanced network deconvolution (BND) applies complex neural network theory to calculate a new matrix without the transitive noise.

**Table 4:** Protein contact prediction tools. The list was compiled from tools described in [140], [129], [141].

**Name**	Method*	**URL**	**Reference**
PSICOV	PSICOV	http://bioinf.cs.ucl.ac.uk/downloads/PSICOV	[142]
GREMLIN	plmDCA	http://gremlin.bakerlab.org	[143]
Freecontact	mfDCA	https://rostlab.org/owiki/index.php/FreeContact	[144]
CCMpred	plmDCA	https://github.com/soedinglab/ccmpred	[145]
FALCON-Contact	clmDCA	http://protein.ict.ac.cn/clmDCA/	[146]
MetaPSICOV	PSICOV	http://bioinf.cs.ucl.ac.uk/MetaPSICOV	[147]
PconsC	PSICOV/plmDCA	http://c.pcons.net/	[148]
BND	BND	http://www.csbio.sjtu.edu.cn/bioinf/BND/	[149]
R2C	SVM	http://www.csbio.sjtu.edu.cn/bioinf/R2C/	[150]
RaptorX	Residual CNN	http://raptorx.uchicago.edu/ContactMap/	[151]
DeepContact	Residual CNN	https://github.com/largelymfs/deepcontact	[152]
DeepCov	CNN	https://github.com/psipred/DeepCov	[153]
SPOT-Contact	Residual CNN/BLSTM	http://sparks-lab.org/jack/server/SPOT-contact/	[154]
ResPRE	CNN	https://zhanglab.ccmb.med.umich.edu/ResPRE/	[155]
TripletRes	Multi-stage residual CNN	https://zhanglab.ccmb.med.umich.edu/TripletRes/	[132]
ResTriplet	CNN	https://zhanglab.ccmb.med.umich.edu/ResTriplet/	[132]
DeepMetaPSICOV	CNN	https://github.com/psipred/DeepMetaPSICOV	[156]
DESTINI	CNN	http://pwp.gatech.edu/cssb/destini	[157]
RBO-Epsilon	CNN	https://compbio.robotics.tu-berlin.de/epsilon	[158]
PconsC4	DCA/CNN	https://github.com/ElofssonLab/PconsC4	[159]
AlphaFold	Residual CNN	https://deepmind.com/	[120]
DeepCDpred	Multi-stage FFNN		[130]
DNCON2	Multi-stage CNN	http://sysbio.rnet.missouri.edu/dncon2/	[160]

*BLSTM: bidirectional long short-term memory neural networks. BND: balanced network deconvolution. CNN: convolutional neural networks. DCA: direct-coupling analysis (clmDCA: composite likelihood maximization DCA. mfDCA: mean-field approximation DCA. plmDCA: pseudo-likelihoods DCA). FFNN: feed forward neural networks. PSICOV: protein sparse inverse covariance analysis. SVM: support vector machines.

## 9 Other modeling challenges

### 9.1 Intrinsically Disordered Proteins (IDPs)

The world of protein folding has one more mysterious – albeit unfolded – tale that is yet to be told (or fold!). IDPs and the intrinsically disordered protein regions (IDPRs) encompass a vague area of protein science with different rules and possibly unique functions. IDPs and IDPRs are commonly present in all living organisms, with a number that is proportional to the complexity of the organism. Nowadays, numbers are speaking about over 1150 IDPs that possess their own folding rules in terms of conventional biophysical concepts, which are thought to constitute the understanding of the world of protein 3D structure, thus breaking the “structure–function” and “lock and key” paradigms [161], [162], [163], [164], [165], [166], [167]. In contrast to globular proteins, IDPs not only lack unique 3D structures during the journey of folding, but they are also unable to settle on just one choice, with an extraordinary spatiotemporal heterogeneity. In other words, while IDPs are jumping between the many structural states, they settle for different periods in every station on the train of free energy map track. In theory, the folding of any part of the IDPs is random and without exact structural homology. These unsynchronized parts react with unique responses to the different environmental changes, which can be understood after knowing that these proteins have relatively flat and simple free energy landscape. This means that they do not have a singular folded state in the free energy landscape with the most distinguished downhill. Hence, IDPs are characterized by reduced informational content in their amino acid sequences due to richness of disorder-promoting residues (Arg, Pro, Gln, Gly, Glu, Ser, Ala, and Lys). Biophysically speaking, this would leave far fewer restraints for the polypeptide to fold and more solvent accessibility; promoting a dynamic structural state [168], [169], [170], [171], [172].

In order to tackle this difficult paradox, state-of-the-art tools are used to decipher structural complexity. The multidimensional NMR can be combined with small-angle X-ray scattering (SAXS), and then processed using advanced computational data integration via molecular dynamics simulations [173]. Other approaches include single-molecule fluorescence resonance energy transfer [174], and atomic-force microscopy [175]. Traditional methods are expensive and time-consuming, especially in the aspects of purifying and crystallizing IDPRs; therefor researchers were holding their hopes on integrating state-of-the-art tools with advanced computational methods [176], [177]. The latter can be divided to three approaches: The first one depends on physicochemical properties and propensity scales (*e.g.*, IsUnstruct tool) [178], [179]. The second one depends on ML techniques (*e.g.*, SPINE-D tool) [180]. The third one combines several predictors so it is called the *meta*-approaches (*e.g.*, Meta-Disorder predictor) [181]. One branch of the *meta*-approaches is a template-based method that depends on homologous known-structure proteins (*e.g.* GSmetaDisorder3D) [182].

The scientific community has recently committed to answering different questions regarding IDPs collaboratively. The critical assessment of protein intrinsic disorder prediction (CAID) is the first fully blind assessment of IDPs predictors, which was obviously inspired by the CASP achievements. CAID addressed two major points in its first edition: Firstly, providing a clear definition of IDPs, and secondly, developing concise strategies to evaluate the performance of prediction methods. Knowing that several editions of the CASP experiment have attempted to tackle the same problems without sufficient results, the CAID was more focused and specialized in this area. CAID experiment has shifted the efforts in providing promising data and in improving the definitions of the boundaries of disordered binding regions [183], [184], [185].

### 9.2 Modeling multiple domains

Nearly 75% of proteins consist of multiple domains (average 2.1 domains in eukaryotes and 1.5 domains in prokaryotes) which are independent structural and evolutionary units that are often reshuffled in genomic rearrangements to form quaternary structures [186], [187]. Understanding the 3D structure of multiple domains proteins can shed the light on various biochemical mechanisms including the role of mutations in driving multiple domains functions and their association with disease [188]. Many studies have been trying to develop refinement tools for multi-domain 3D structure analysis based on data collected from SAXS [189], [190], [191], and cryo-EM density maps [192]. Homology modeling-based programs for prediction and/or assembly of multidomain structure include *ab initio* domain assembly (AIDA; http://ffas.burnham.org/AIDA/) [186] and Multidomain Assembler (MDA package in UCSF CHIMERA) [187].

## 10 Future directions

To this day, the deterministic search algorithms mentioned in the introduction have worked hand-in-hand with experimental methods. Preliminary information from spectroscopic methods, crystallography and even limited number of atomic contacts in NMR experiments are used these days to optimize deterministic algorithms of molecular dynamics and homology modeling to produce cheaper yet more accurate folding predictions. It is known that excluding large sub-spaces of c-space allows for the detailed scan of larger sized molecules [193]. Cryo-EM has broken significant barriers recently, thus bringing detailed atomistic resolution (up to the level of hydrogen atoms) to the study of protein complexes, virus particles and sub-cellular organelles.

The lesson learned from deep learning of contact maps in recent years highlights the need for adding more layers of information and processing in future trends in homology modeling. Taking the analogy of onion layers, homology modeling prediction accuracy has advanced each decade through adding new layers of information and processing. This has been carried out by introducing multiple templates and information of secondary structure predictions, then by optimizing *ab initio* loop modeling, by employing backbone-dependent rotamer libraries for side-chains addition, and finally by developing deep learning contact maps. The evaluation procedure itself evolved over time. It is very hard to predict what the next layer of the onion would be or what deep impact it can lay upon the field. However, we can at least warn that currently used programs should be written in a format that accepts new additions at any step of the homology process. In this review, we have neglected the developments in computer hardware, which can make costly computations feasible in the near future. There has been several promising strategies that may enhance 3D structure predictions, such as chi1 angle prediction, new structural annotations, solvent accessibility studies, molecular simulations, and hybrid *ab initio*-homology modeling methods.

Even by using the same datasets, the process of deep learning will continue to improve. One very optimistic recent view sees that “by the end of this century, it is expected that computers will have the power to train neural networks with as many neurons as the human brain” [194]. This view is supported by recent developments in this field. In the future, deep learning in dynamic environment will learn through reward-based reinforcement neural networks, which brings continuity to the prediction process. Separation of the storage from computation will ultimately optimize deep learning. Recurrent neural networks that can control reading and writing from external memory are under development. ML became more feasible after development of Graphics Processing Units and currently deep learning will too become feasible through development of Tensor Processing Units (TPUs), which can perform computations in more dimensions. TPUs are specialized integrated circuits developed by Google, LLC (Mountain View, CA, USA) for applications in ML.

In conclusion, it is evident that the gradual and recent integration of CI and AI has played a significant role in the development of homology modeling accuracy. CI contributed to the development of evaluation methods and the addition of new steps in homology modeling. AI contributed to the data processing and prediction efficiency through contact maps ML. If we perceived the homology modeling as a process of consequent steps executed by independent modules, it is very clear that the accuracy of the homology models was enhanced by introducing new modules, and also by improving current modules. It is hoped that such specialization in modeling tools development will make it possible to customize and test combinations of modules in the future. Here, CI and AI will play great role in integration of different resources for more efficient modeling.

## Author contributions

The manuscript was written through contributions of all authors. All authors have given approval to the final version of the manuscript.

## CRediT authorship contribution statement

**Tareq Hameduh:** Writing - original draft. **Yazan Haddad:** Conceptualization, Writing - original draft. **Vojtech Adam:** Supervision, Funding acquisition, Writing - review & editing. **Zbynek Heger:** Supervision, Conceptualization, Writing - review & editing.

## Declaration of Competing Interest

The authors declare that they have no known competing financial interests or personal relationships that could have appeared to influence the work reported in this paper.
